# Impact of Three Different Dehydration Methods on Nutritional Values and Sensory Quality of Dried Broccoli, Oranges, and Carrots

**DOI:** 10.3390/foods9101464

**Published:** 2020-10-14

**Authors:** Xanyar Mohammadi, Yuhao Deng, Golshan Matinfar, Anika Singh, Ronit Mandal, Anubhav Pratap-Singh

**Affiliations:** 1 Food, Nutrition and Health Program, Faculty of Land and Food Systems, The University of British Columbia, Vancouver, BC V6T 1Z4, Canada; xanyar91@mail.ubc.ca (X.M.); dengyuhao19951015@gmail.com (Y.D.); anika.singh@ubc.ca (A.S.); ronit.mandal@ubc.ca (R.M.); 2 Department of Food Science and Technology, National Nutrition and Food Technology Research Institute, Shahid Beheshti University of Medical Sciences, Tehran 1981619573, Iran; matinfargolshan@gmail.com

**Keywords:** radiant energy vacuum drying (REV-drying), freeze drying, air drying, broccoli, orange, carrot, sensory quality, accelerated shelf life

## Abstract

Radiant Energy Vacuum (REV)-dried broccoli, oranges, and carrots prepared by the optimal drying protocols determined in this study were compared to the freeze-dried and air-dried samples based on the nutritional values before and after drying. An accelerated shelf-life study for REV-dried broccoli, oranges, and carrots was also conducted. For all the samples, REV drying significantly shortened the processing time. The REV-dried samples had much higher retention of the nutritional values (vitamin C, β-carotene) compared to the conventional air-drying process, and the values were also competitive to those of the freeze-dried samples. Although freeze-drying resulted in the best rehydration property, the REV-dried samples still earned the highest scores in the sensory test. In the accelerated shelf-life study conducted on the REV-dried samples, the moisture content and water activity stayed at the same level, but the nutritional values showed a downward trend. The sensory properties fluctuated in the shelf-life but still gained positive feedback from the panelists. Moreover, the testing method for β-carotene content was uniquely designed in this project and could be a semi-quantitative method to refer to.

## 1. Introduction

As one of the most important sources of essential nutrients, such as different kinds of vitamins and fiber, the consumption of fruits and vegetables is becoming more and more common in people’s dietary habits [1]. Today, there is a broad observation that human health can be influenced by fruit and vegetable phytochemicals. Specifically, phytochemicals with antioxidant characteristics provide health-protective results of fruits and vegetables. In fact, oxidative stress in disease may be alleviated by antioxidants [2].

Broccoli, which is one species of Brassicaceae vegetables, was found to be able to lower the possibility of developing cancer due to its high content of different kinds of bioactive compounds, especially vitamin C [3]. Orange is also rich in vitamin C and considered to be one category of citrus fruit of high physiochemical and nutritional quality [4]. Carrot is regarded as a highly nutritious vegetable for its high amount of carotene content, especially β-carotene, which is of antioxidant property and is able to show anticancer activities [5,6]. As exhibited in United States Department of Agriculture (USDA) national nutrient database, the moisture contents of fresh broccoli, oranges, and carrots are all at high values, approximately 89% for broccoli, 86% for oranges, and 88% for carrots [7,8,9]. Due to the high moisture content, the high nutrient levels, the existence of sugar, protein, etc., as a nutrient source for microorganisms, storage conditions, etc., all these three categories of fruits and vegetables are distinguished as highly perishable products [1]. Dehydration has been applied for preserving fruits and vegetables for centuries and is still one of the most common preservation methods for these products [10,11]. By lowering the water activity and getting rid of most of the water content, dehydration would help to keep products from spoiling in a relatively longer shelf-life. However, the choice of the dehydration method can have a large impact on the quality attributes of the products, ranging from the visible color and texture to the invisible nutrient values [11].

Air-drying is one of the most popular dehydration methods in the food industry. By applying hot air to the surface of the food products, the water content of the products is significantly decreased to an acceptable level which, in turn, maintains microbiological safety. However, the process can also be time-consuming and generally, the thermal treatment for a prolonged period also results in the loss of the quality in products which are sensitive to high temperature. These products also tend to shrink in shape and the nutritional values are also lowered to a great extent [12].

Freeze-drying is another dehydration method usually accepted by food companies to process high-moisture food products. It is usually considered the gold standard of drying operations. By applying vacuum under a freezing temperature, the food products are kept frozen during the process and the water content is lowered through continuous sublimation. Due to the low temperature during processing, the shape of the final products is retained, and the nutritional values could also be maintained. Although the treatment benefits from all these advantages, the whole process can be time-consuming and expensive [13]. Depending on the products and the process parameters, the final flavor of some sensitive products gets deteriorated [14].

Radiant Energy Vacuum (REV) dehydration is one kind of advanced rapid, low-temperature drying method. When the REV is applied to the food products, the microwave energy under vacuum is applied and absorbed by the moisture in the food products which, in turn, creates a large inside vapor pressure differential between interior and exterior of product. Under the vacuum condition, the vapor inside the food is continuously transferred to the surface, where a much lower pressure than the interior parts of the food products is expected. In this way, the water content in the food products is lowered to an acceptable level in a relatively short time without causing severe damage to the shape and the texture of the products in comparison to conventional air drying. Moreover, the relatively lower processing temperature would also help to maintain more nutrients than conventional thermal treatment [15].

The purpose of our research was to determine the impact of three different dehydration methods, air drying, freeze drying, and REV drying, on the nutritional values, sensory characteristics, and shelf life of different vegetables—broccoli, oranges, and carrots.

## 2. Materials and Methods 

### 2.1. Sample Preparation and Drying Processes

**Figure 1:**
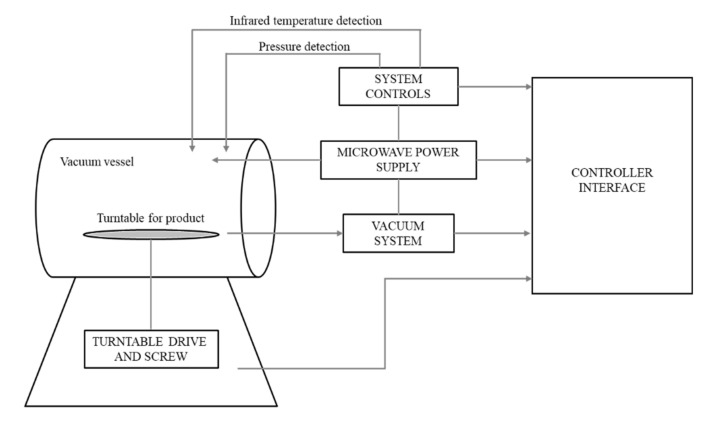
Schematic diagram of the Radiant Energy Vacuum (REV)-drying process.

Fresh broccoli florets, Navel oranges, and carrots were bought from a local market and stored in refrigeration before pretreatment. For air-drying of the samples in this study, the Harvest Saver Tray Dryer Commercial Dehydrator Systems, Inc. (CDS, Eugene, OR, USA) was used for all samples. In the freeze-drying process, the Labconco FreeZone 6L freeze drier (Kansas City, MO, USA) was used for all the samples. Also, the REV drying process for all samples in this study was done in the vacuum-microwave drying machine uniquely designed by EnWave Corporation (Vancouver, BC, Canada) with the maximum power level to be 2 kW. A schematic diagram of this equipment is shown in Figure 1. The system consisted of a vessel with a turntable where the product was kept and vessel door was shut. The microwave power and vacuum level in the vessel were switched on and controlled by a controller interface.

#### 2.1.1. Drying of the Broccoli

Fresh broccoli florets were steam blanched for 5 min and immediately placed in a −24 ℃ freezer to lower down the temperature of steam blanching. The initial weight of the steam blanched florets used for REV drying was 2 kg. The vacuum applied was 25 ± 2 Torr (3.333 ± 0.267 kPa), and the temperature was set to be 65 ℃. The rotation speed was set to be 8 rpm. The power levels used were 2 kW, 1 kW, and 600 W, with the processing time set to be 2070, 3240, and 2400 s, respectively. The time and power levels were determined by the company based on preliminary trials. Then, 1 kW REV processed samples were later analyzed for all properties. The total energy consumed was 2.443 kWh. For the freeze-drying of broccoli, 320 g of broccoli florets was placed in the freeze-dryer. The vacuum applied was 0.009 Torr (1.2 Pa), and the temperature during the process was −85 ℃. The total processing time was 88 h. For air-dried Broccoli, the initial weight of the broccoli florets was 320 g. The temperature was set to be 70 ℃ and the time for processing was 4 h.

#### 2.1.2. Drying of the Oranges

Orange samples were sliced to 3.5 mm thickness and stored in the refrigerator before drying. In REV-drying, 2 kg of orange slices was first air-dried at a temperature of 70 °C to a moisture content of 40% and stored in the refrigeration overnight for equilibrium. Coconut oil, with the amount to be 2% of the total weight, was added to the orange slices right before drying to prevent orange slices from sticking together. The process parameters were set as same as the drying of broccoli, and the power levels used were 1 kW and 600 W, with the processing time set to be 400 s and 600 s. Then, 1kW REV processed samples were later analyzed for all properties. The total energy consumed was 0.229 kWh. For the freeze-drying of the orange samples, the initial weight of the orange slices was 340 g. Applied vacuum and temperature were the same as drying broccoli, and the total processing time was 96 h. For air-dried oranges, the initial weight of the samples was 320 g. The temperature was set to be 70 ℃ and the time for processing was 3 h and 10 min.

#### 2.1.3. Drying of the Carrots

Both ends of the carrots were cut, and the rest was sliced to 3 mm. Afterward, the samples were water blanched for up to 15 min and kept frozen in the −24 ℃ freezer right after blanching. For REV-dried carrots, the initial weight of the carrot slices was 1.5 kg. Coconut oil, with the amount to be 2% of the total weight, was added right before drying. The process parameters were set as same as the drying of the other two products, and the power levels used were 2 kW, 1 kW, and 600 W, with the processing time set to be 1500, 2000, and 2620 s, respectively. Then, 1kW REV processed samples were later analyzed for all properties. The total energy consumed was 1.794 kWh. For freeze-dried carrots, the initial weight of the carrot slices was 300 g. Applied vacuum and temperature were the same as two other products, and the total processing time was 96 h. Lastly, for air-dried carrots, the carrot slices′ initial weight was 300 g. The temperature was set to be 70 ℃ and the time for processing was 1 h and 35 min.

### 2.2. Methods

#### 2.2.1. Moisture Content

A vacuum oven (Thermo Fisher Scientific, USA) was used to determine the moisture content of all the samples before and after the treatments. Approximately 1.5 g of food samples was accurately weighed in an aluminum foil vessel. The weight of both the empty vessel and the total weight were recorded. Then, the samples were placed in the vacuum oven for up to 24 h at the temperature of 90 °C and weighed again after drying. All the measurements were conducted in triplicate to ensure the validity of the data. The moisture content could be calculated by Equation (1).

(1) $$Moisture\;content=\frac{Weight\;\left(Wet\;sample+vessel\right)-Weight\;\left(Dry\;sample+vessel\right)}{Weight\;\left(Wet\;sample+vessel\right)-Weight\;\left(Empty\;vessel\right)}\times 100,$$

#### 2.2.2. Water Activity

A water activity analyzer (Aqualab Pre, Meter Group Inc., Pullman, WA, USA) was used to determine the water activity of all the samples before and after the treatments. The water activity analyzer was turned on at least 20 min before using to warm up. A validation test was conducted each time before measuring. Afterward, 1-2 g of fresh and dried samples was smashed and put into the water activity analyzer. The readings were recorded. All the tests were conducted in three biological replicates. 

#### 2.2.3. Vitamin C

Vitamin C measurement was carried out by following a method described by Santos et al. [16] for broccoli and orange samples after drying and over the accelerated shelf-life study. Fresh products were frozen in a −80 ℃ freezer for 15 min before blending. Both fresh and dry products were blended into a fine powder and a second round of blending was needed for fresh broccoli. Blended samples were mixed with 40 mL 1% (*w/v*) metaphosphoric acid. Then, the samples were stored in refrigeration for 1 h and immediately after that the mixtures were centrifuged. The absorbance of the sample solution was measured at 515 nm by a Thermo Fisher Scientific Evolution 60s Ultraviolet-Visible (UV-vis) Spectrophotometer (Thermo Electron Scientific Instruments LLC, Madison, WI, USA). Then, an ascorbic acid standard curve was used to quantify the amount of Vitamin C in the samples. 

#### 2.2.4. β-Carotene

The method for measuring the β-carotene was a method described by Prakash et al. [17] and the value was measured for carrots before and after the drying process. Briefly, approximately 0.1–0.5 g of dried and fresh samples was ground thoroughly to a powder form by a blender. Then, the samples were extracted in a falcon tube by a 25 mL mixture solution consisting of 7.5 mL of acetone and 17.5 mL of petroleum ether. The extract was then filtered with Whatman No.1 Filter Paper. The extraction was repeated with the acetone/petroleum ether mixture solution (3:7, *v/v*) until the residue on the filter paper turned colorless. Afterward, the filtrates were transferred to a separatory funnel. A total of 50 mL of distilled water was added in the separatory funnel to wash the solution. After 5 min of standing time, the water phase was discarded and around 0.5 g of anhydrous sodium sulfate was added to remove the remaining water. After another 5 min of standing time, the petroleum ether phase was transferred to a 50 mL volumetric flask and brought to the volume of 50 mL by adding petroleum ether. The absorbance of the sample solution was measured at 452 nm by a Thermal Fisher Scientific Evolution 60s UV-visible Spectrophotometer To draw a standard β-carotene curve, 0.008 mg/mL β-carotene solution was diluted to 100%, 80%, 60%, 40%, 20%, and 0% of the original concentration by adding petroleum ether. Then, β-carotene solutions of different levels of concentration were measured at 452 nm by the spectrophotometer. The standard curve was finally obtained by graphing the concentration (mg β-carotene/mL) versus absorbance.

#### 2.2.5. Sensory Evaluation

The sensory evaluation of all the samples was carried out by eight trained and experienced panelists from EnWave Corporation and The University of British Columbia. A five-point hedonic sensory evaluation was used in the project and the scores ranged from −2 to 2 (dislike very much to like very much). The sensory attributes used in the project were appearance, aroma, texture, flavor, and overall quality. All the samples tested for sensory attributes were numbered randomly to minimize bias.

#### 2.2.6. Rehydration

The rehydration potential of the dried samples of all three treatments was evaluated by immersing around 1–2 g of the samples in 30 ℃ water. The weight of the samples at 0, 2, 4, 6, 8, and 10 min was recorded.

#### 2.2.7. Drying Efficiency Analysis

Drying is an energy intensive process, which necessitates the study of efficiency of drying process. The drying efficiencies were calculated for REV-drying based on the parameters: specific moisture extraction rate (SMER), specific energy consumption (SEC), and exergy efficiency (η_ex_). The different power levels mentioned in Section 2.1 were used in the analysis.

The SMER was calculated as the ratio of moisture evaporated during drying to the associated energy consumption using Equation (2):

(2) $$\mathrm{SMER}=\frac{\mathrm{Moisture}\;\mathrm{removed}\;\left(\mathrm{kg}\right)}{\mathrm{Energy}\;\mathrm{comsumed}\;\left(\mathrm{kWh}\right)\;}=\frac{m_{w}}{E_{t}},$$

where $E_{t}$ = energy consumed during process (kWh), calculated as the microwave energy used: microwave power × drying time (t).

The SEC was calculated as the ratio of energy consumed (MJ) to dry a kg product as per Equation (3).

(3) $$\mathrm{SEC}=\frac{\mathrm{Energy}\;\mathrm{comsumed}\;\left(\mathrm{MJ}\right)}{\mathrm{Moisture}\;\mathrm{removed}\;\left(\mathrm{kg}\right)\;}=\frac{E_{t}}{m_{w}},$$

In addition, the exergy analysis was done. Exergy can be defined as the “maximum work that can be derived from a system or a stream of matter or energy as that system comes to equilibrium with a reference environment” [18]. The exergy efficiency (η_ex_) was calculated as the ratio of rate of exergy used in the moisture removal to the total power consumption (Equation (4)):

(4) $$\eta_{\mathrm{ex}}=\frac{\mathrm{Rate}\;\mathrm{of}\;\mathrm{exergy}\;\mathrm{used}\;\mathrm{for}\;\mathrm{moisture}\;\mathrm{removal}\;\left(W\right)}{\mathrm{Power}\;\mathrm{consumption}\;\left(W\right)}\times 100=\frac{{\mathrm{Ex}}_{\mathrm{evap}}}{P_{t}}\times 100,$$

where $Ex_{evap}$ = rate of exergy used for moisture removal calculated by Equation (5):

(5) $${\mathrm{Ex}}_{\mathrm{evap}}=\left(1-\frac{T_{o}}{T_{p}}\right)m_{w}^{\prime }\lambda_{\mathrm{wp}},$$

where $T_{o}$ = ambient temperature (20 ℃); $T_{p}$ = product temperature (based on the saturation temperature at the saturation pressure [3.333 kPa], taken as 25.85 ℃); $m_{w}^{\prime }$ = rate of moisture removal, kg/s; $\lambda_{\mathrm{wp}}$ = latent heat of vaporization of product (calculated by multiplying moisture content of the product with the latent heat of vaporization of water), kJ/kg. 

#### 2.2.8. Storage Study

An accelerated shelf-life study was conducted for REV-dried products to monitor the changes in the quality parameters during a harsh temperature. All samples for the shelf-life study were packaged in aluminum foil packing bags and stored in an incubator. The incubator parameters were set to be 35 ℃ for the temperature and 25% for the humidity. Parameters, including moisture content, water activity, and vitamin C, were measured after 0, 7, 14, 28, and 42 days of incubation. Kinetic modeling of vitamin C degradation was carried out based on the first-order model. In addition, sensory parameters, including appearance, aroma, texture, flavor, and overall quality, were also evaluated according to the explained prior method to observe the changes in these parameters. 

### 2.3. Statistical Analysis

The experimental data were processed statistically by IBM SPSS Statistics v. 27 (New York, NY, USA) and Microsoft Excel 365 (Microsoft Corp., Redmond, WA, USA). The significance of differences between treatments was determined by one-way ANOVA and Duncan’s multiple range test (*p* ≤ 0.05). Statistical parameters such as determination coefficient and *R*^2^ were used to evaluate the goodness of fit.

## 3. Results and Discussion

### 3.1. Moisture Content and Water Activity before and after Drying

The moisture content and water activity of fresh broccoli, orange, and carrot in comparison to dried products of all three treatments are shown in Table 1 below.

In dried products, the two most crucial aspects to be analyzed for ensuring food safety and quality of the products are the residual moisture content and water activity. The moisture content of dried fruits and vegetables must be low, and the maximum water activity must be 0.6 or lower [19]. As shown in Table 1, a significant variation in water activity and moisture content can be seen. However, all three dehydration methods had a marked decrease in moisture content and water activity to a safe point, although the moisture content and water activity of the freeze-dried products were lowest among all three treatments due to the porous structure and lowest case hardening [20].

### 3.2. Nutritional Value before and after Drying

**Figure 2:**
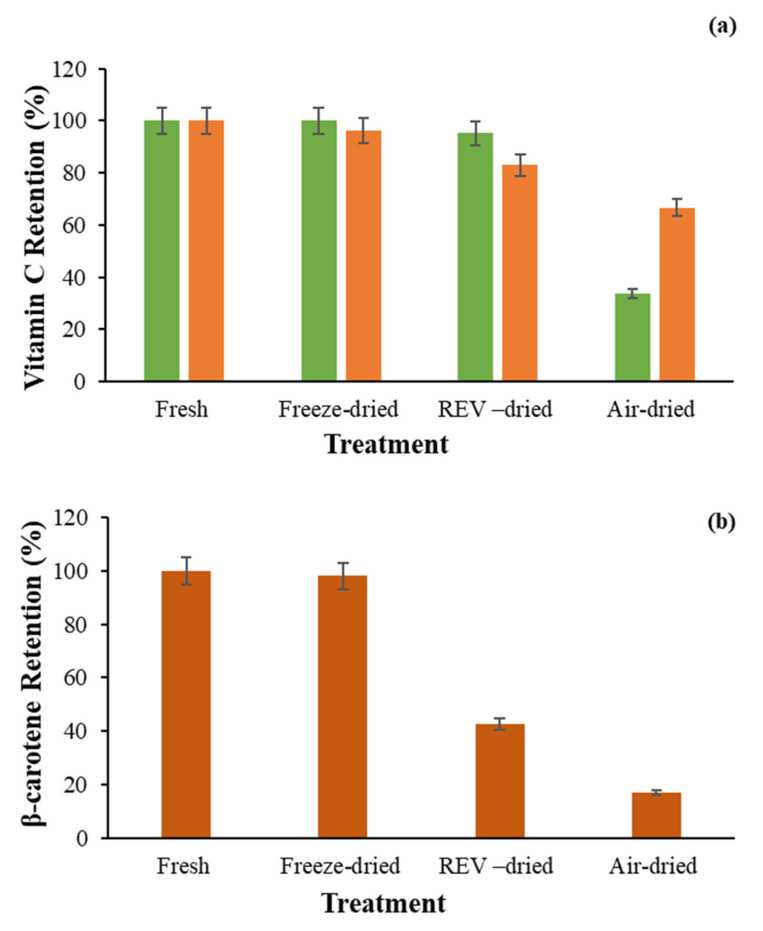
(**a**) Retention of Vitamin C in broccoli (▪) and oranges (▪); (**b**) Retention of β-carotene in carrot; Radiant Energy Vacuum dried (REV-dried).

Generally, the overall nutritional quality of processed food is evaluated by the retention of ascorbic acid. This nutrient is highly susceptible to degradation, as it is highly water-soluble, sensitive to heat, and oxidation condition (oxygen, pH, and metal ions) [21]. The vitamin C contents of fresh and dried broccoli and orange were shown in Table 1. The values of fresh broccoli, freeze-dried broccoli, REV-dried broccoli, and air-dried broccoli were 3.79, 3.77, 3.61, and 1.27 mg ascorbic acid/gram of dry sample, respectively. As reported by USDA, fresh broccoli will have approximately 0.892 mg ascorbic acid/gram of fresh samples at a moisture content of 89%, which is roughly 7.60 mg ascorbic acid/gram of dry sample at a moisture content of 6% [22]. Thus, the steam blanching pretreatment process resulted in an approximately 50.2% decrease in the total vitamin C content in fresh broccoli. This is in agreement with results seen by Maharaj and Sankat [23]. As for the drying process, the REV-drying resulted in a 4.6% decrease in the total vitamin C content, while the air-drying resulted in a 66.4% decrease in the total vitamin C content (Figure 2a).

The vitamin C content of fresh oranges, freeze-dried oranges, REV-dried oranges, and air-dried oranges was 3.97, 3.82, 3.29, 2.65 mg ascorbic acid/gram of dry sample, respectively. According to USDA, fresh navel oranges will have approximately 0.592 mg ascorbic acid/gram of fresh samples at a moisture content of 81%, which is about 2.9 mg ascorbic acid/gram of dry sample at a moisture content of 6% [24]. The values for fresh and dehydrated oranges in this study are all higher than the suggested value, which may be attributed to different genotypes of the oranges, the different locations of harvesting, the sunlight difference, and the individual differences within the oranges [25]. The REV-drying resulted in a 16.9% decrease in the total vitamin C content, while the air-drying resulted in a 33.3% decrease in the total vitamin C content.

The values of β-carotene content were measured before and after drying for fresh carrots and dehydrated carrots. The details are presented in Table 1. The results showed that the fresh carrots, the freeze-dried carrots, REV-dried carrots, and the air-dried carrots had 3.03, 2.98, 1.29, and 0.52 mg β-carotene/gram of solids, respectively. During the drying process, the REV-drying resulted in a 57.5% reduction in the total β-carotene content, and the conventional air-drying process resulted in an 82.9% reduction, which meant the REV-drying method could better retain the β-carotene content compared to the conventional air-drying method (Figure 2b).

Factors associated with carotene loss are heat, oxygen, and lipoxygenase activity [26]. The reason for carotene loss in hot air drying can be explained in this way, that carrots exposed to high-temperature air lose their color as their highly unsaturated carotenoid is oxidized by lipoxygenase, which has greater activity at the high temperature, and isomerization of the *trans*-carotenoids to the less colored *cis*-forms by heating [27]. The result for lower carotene degradation in REV-drying is because the oxidation responsible for the degradation of the carotene is considerably decreased because of faster dehydration and lower drying time and limited oxygen available during the process [26]. The freeze-dried carrots had the highest value in the β-carotene content as well as the retention rate, which is because of low-temperature treatment, low lipoxygenase activity, and less oxygen available. In another study conducted by Lin et al. [14], the value of the REV-dried carrots in this project was similar to their measurement, which showed REV-dried carrots to have proximately 1.153 mg β-carotene/g of solids. Although the method used in this project was uniquely designed and is more cost-effective, the results obtained can only be said to be semi-quantitative. In raw carrots, the main carotenoid varieties are β-carotene, α-carotene, and lycopene, among which β-carotene only accounts for half of the total amount [28]. However, when doing the extraction with acetone and petroleum ether, this colored lipid-soluble α-carotene and lycopene may also be extracted, which may, in turn, result in a higher reading in the absorbance. Thus, it is possible that the β-carotene contents detected in this project were higher than the actual number. If a more accurate reading were needed, other methods, such as high-performance liquid chromatography (HPLC), might have to be referred to.

### 3.3. Rehydration Property

**Figure 3:**
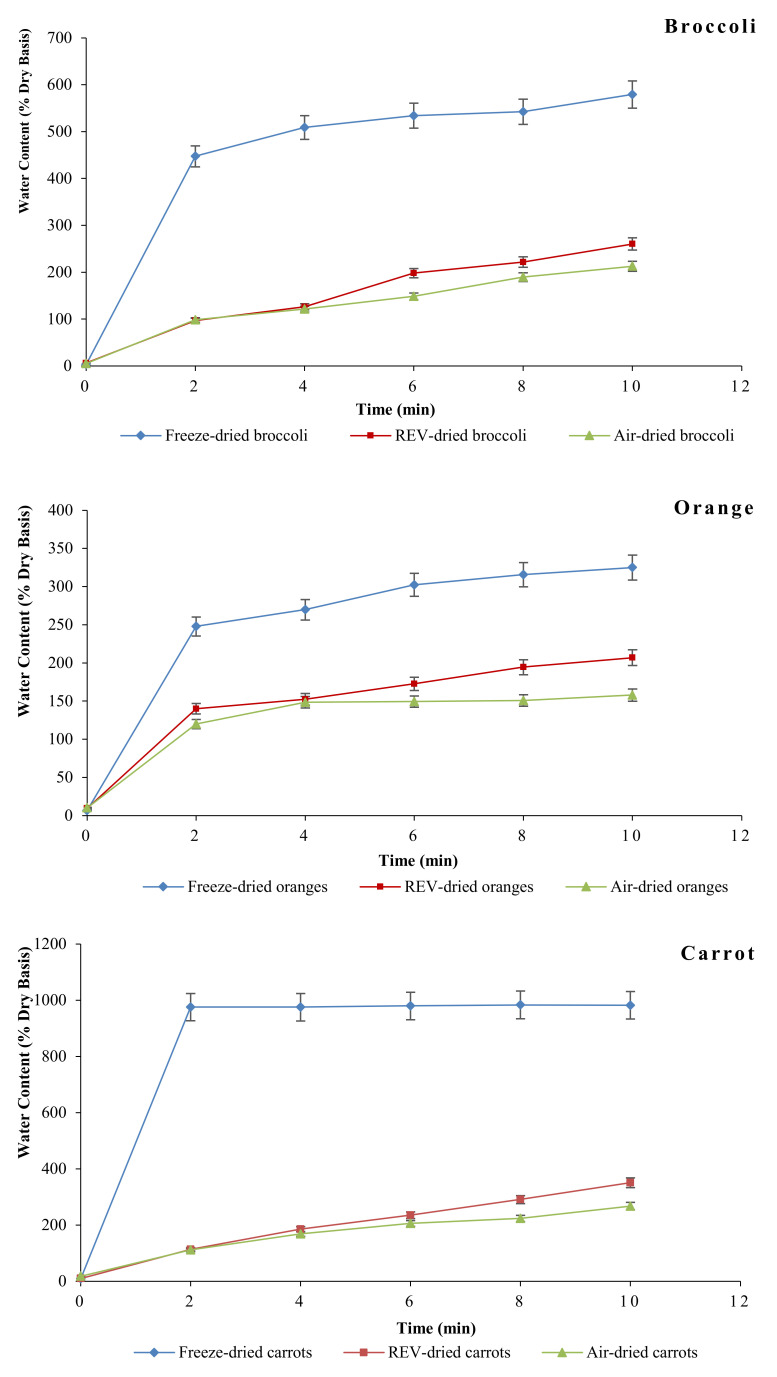
Rehydration curves of REV-dried, freeze-dried, and air-dried products at 30 ℃.

The rehydration properties of dehydrated products of all three treatments are exhibited as changes in the water content (dry base) over the 10-min rehydration test in a 30 ℃ water bath. The details are shown in Figure 3 below. As could be seen from the graphs, the freeze-dried products had the highest potential of rehydration, while the air-dried ones had the lowest. Also, at the beginning of the rehydration, there was a higher rate for all the products; however, it was more evident in freeze-dried products. These results agree with other studies and may be attributed to the highly porous and less dense structure caused by freeze-drying treatment [14,29]. Moreover, by comparing REV- and air-dried products’ rehydration curve, it can be seen that REV-dried products have significantly higher rehydration capacity (*p* ≤ 0.05), and the reason for this is the more open and spongy structure in REV products as a result of vapor expansion during internal water vaporization in the REV process [30].

### 3.4. Sensory Evaluation

**Figure 4:**
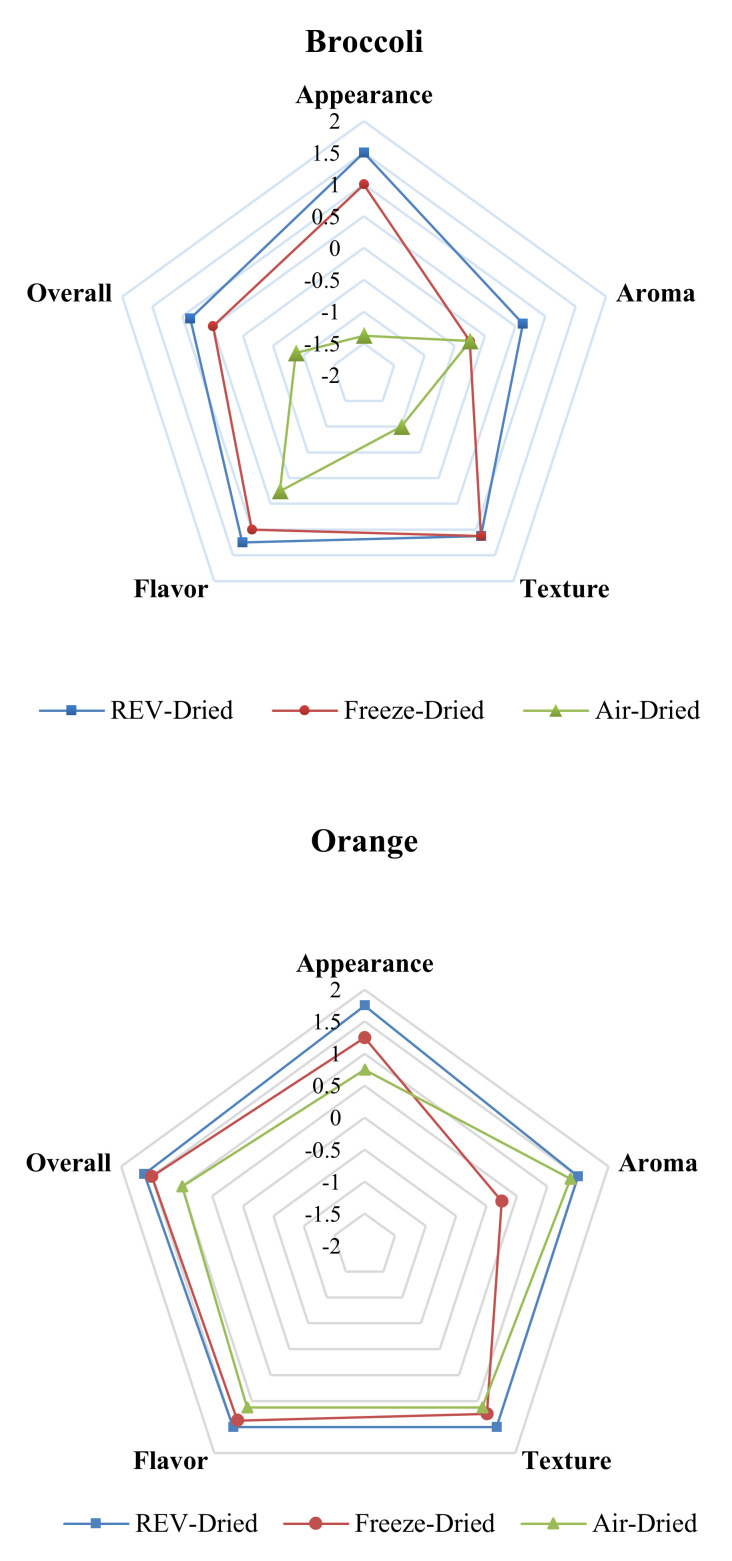

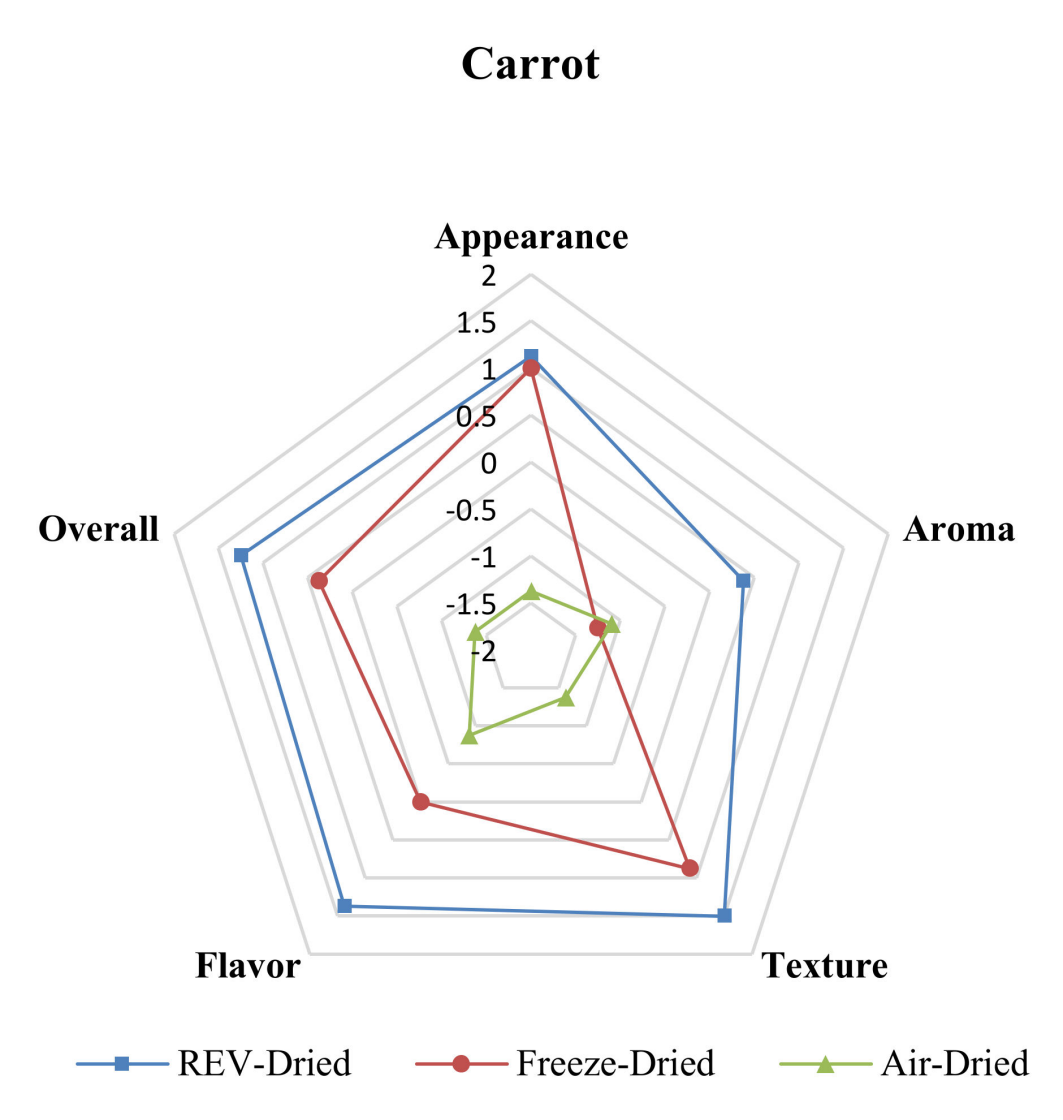
Cobweb diagram of five sensory attributes of REV-Dried, freeze-Dried, and air-Dried products based on panelists′ rating.

**Figure 5:**
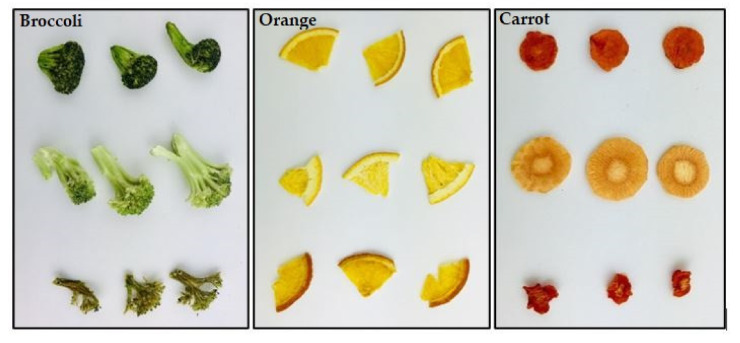
Broccoli, orange, and carrot immediately after drying in the order of the up to down representing REV-dried, freeze-dried, and air-dried.

The sensory evaluation results of REV-dried, freeze-dried, and air-dried products are shown in Figure 4 below. The mean scores of sensory attributes, including appearance, aroma, texture, flavor, and overall acceptability are shown on a scale of −2 to 2 for all the types of dried products on a cobweb plot. The REV-dried products scored the highest in all the sensory attributes except for the texture in broccoli; also, the air-dried ones received the lowest except aroma. The freeze-dried products gained positive scores from the panelists for all the attributes except the aroma, and this may be attributed to the significant loss of volatiles during the freeze-drying process [14]. The visual differences between these dried products with three different drying methods is shown in Figure 4 and it is clear that the air-dried products experienced a significant change in appearance and color. Furthermore, heat treatment in REV- and air-drying processes can enhance the aroma of the products [31]. The sensory evaluation test indicated that the REV-drying led to a better taste and optical property compared to freeze-drying and air-drying, and this result is in agreement with previous studies [30,31]. The broccoli, orange, and carrot immediately after REV-drying is shown in Figure 5. 

### 3.5. Drying Effectiveness Analysis

The drying effectiveness for REV-drying was analyzed based on the values of SMER, SEC, and η_ex_. The values of these SMER decreased with the applied microwave power in the REV-drier for all the vegetables. The decrease in SMER with microwave power was due to the removal of comparatively less moisture as the power increased. A similar trend was observed with the η_ex_, which decreased with increasing power. This is in accordance with the fact that the drying process was more efficient at a lower power of 600 W than higher powers. In a study by Pratap Singh et al. [18], after drying brewers’ spent grains at 250 W, η_ex_ of around 25% was obtained. The values of η_ex_ were greater for the broccoli compared to oranges and carrots. The SEC values increased, contrastingly, with the microwave power. The values of SMER, SEC, and η_ex_ are given in Table 2.

### 3.6. Accelerated Shelf-Life Study of REV-Dried Products

#### 3.6.1. Moisture Content and Water Activity

**Figure 6:**
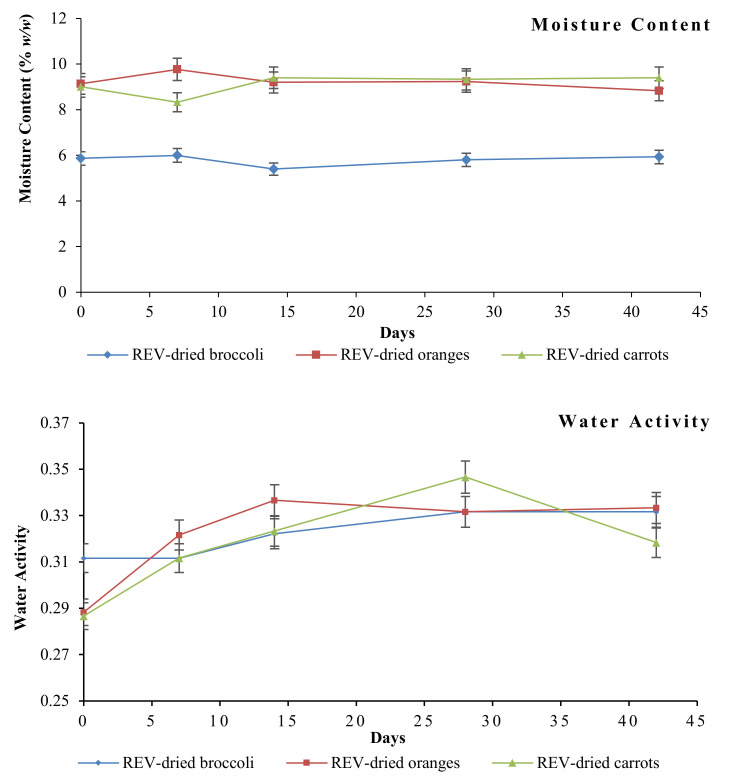
Changes of moisture content and water activity of REV-dried broccoli, orange, and carrot over the accelerated shelf-life.

The moisture content and water activity of the REV-dried broccoli, orange, and carrot were tested on days 0, 7, 14, 28, and 42 after starting the experiment. The trend of changes is shown in Figure 6. As seen from the graphs, the moisture content and water activity of the REV-dried products stayed stable over the accelerated shelf life with no significant variation. This indicates that the products were properly sealed and stored, no extra moisture invaded into the package.

#### 3.6.2. Vitamin C

As for vitamin C content, the analyses were performed for REV-dried broccoli and orange on days 0, 7, 14, 28, and 42 after starting the experiment. Based on several studies that have been done by researchers before, the degradation of vitamin C follows a first-order reaction model (Equation (6)) [32,33,34]:

(6) $$C_{t}=C_{o}\exp\;\left[-\mathrm{kt}\right],$$

where t = reaction time, day; k = rate constant, s^−1^; $C_{t}$ = concentration at time t; $C_{o}$ = initial concentration. 

**Figure 7:**
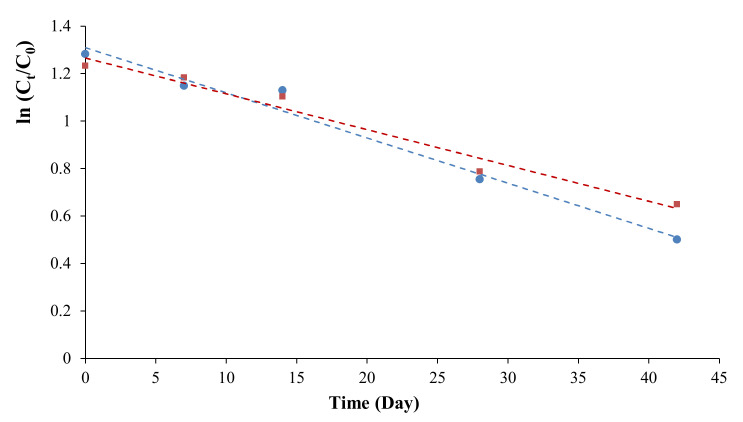
Linearized plot of ln($C_{t}$/$C_{o}$) vs time (Day), for REV-Dried broccoli (•) and orange (▪).

The reaction rate constant k was calculated by using linear regression to plot the natural log (ln) of the ratio of the vitamin C remaining at different days. As can be seen in Table 3 and Figure 7, our results are in good correlation with the first-order reaction model (*R*^2^ > 0.97). Based on the calculated k and half-life $t_{1/2}$, it can be seen that the deterioration rate of vitamin C in the orange was relatively lower than that of broccoli and the more stable form of vitamin C under an acidic condition like oranges may be the reason [35]. In this accelerated shelf-life study, the temperature used was 35 ℃ instead of ambient temperature. Several models demonstrate the effects of heat on the deterioration rate of products during the shelf life, among which Arrhenius equation is the one often referred to [36]. As described in Arrhenius, the relationship between the deterioration rate and the effects of temperature can be stated using Equation (7):

(7) $$k=k_{o}\exp\;[-\frac{E_{a}}{\mathrm{RT}}],$$

where k = constant of the deteriorative rate, s^−1^; $k_{o}$ = Arrhenius factor, which is independent of temperature, s^−1^; $E_{a}\;$ = activation energy, J/mol; R = universal gas constant, J/mol-K; T = absolute temperature, K.

As can be seen from the equation, as the storage temperature increases, the deterioration rate of the products will also be accelerated. At the elevated temperature, which was 35 ℃ in this case, the deterioration rate will be accelerated to around four times compared to storage in the ambient temperature, which may be the reason for the decrease in the vitamin C content [36].

#### 3.6.3. Sensory Evaluation During Accelerated Shelf-Life Study

**Figure 8:**
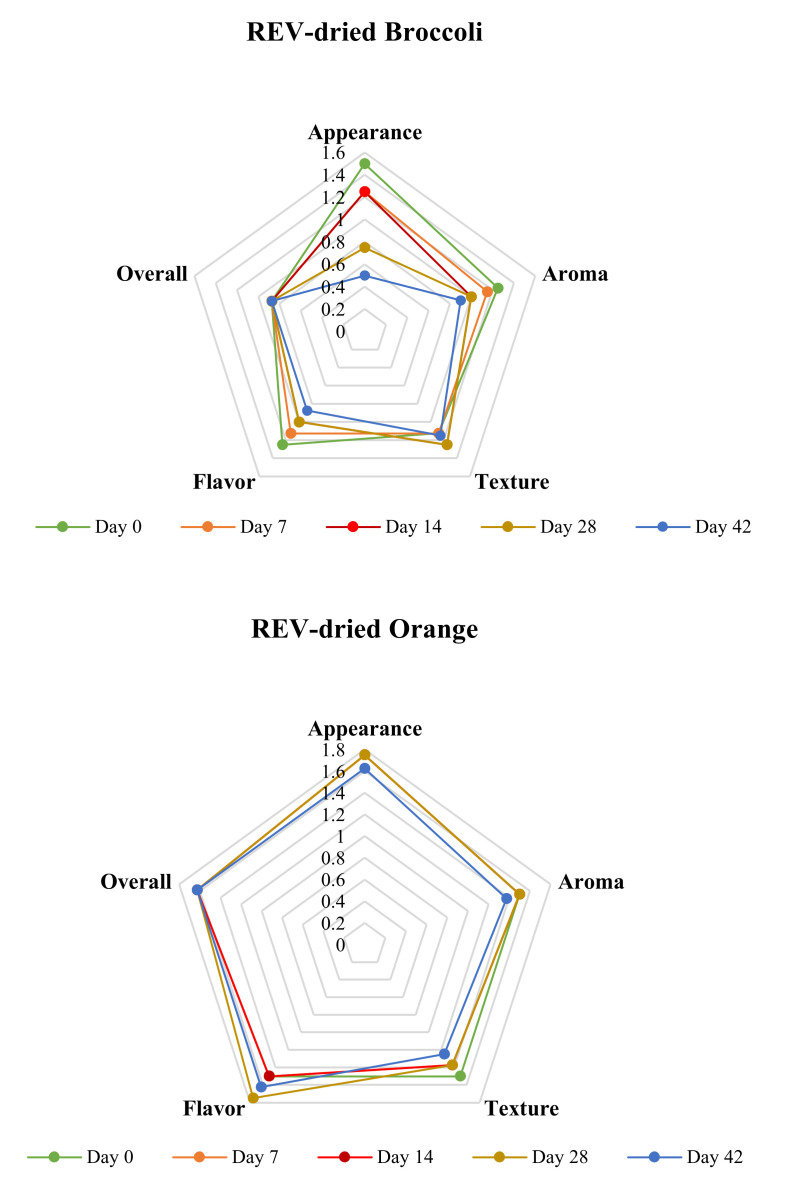

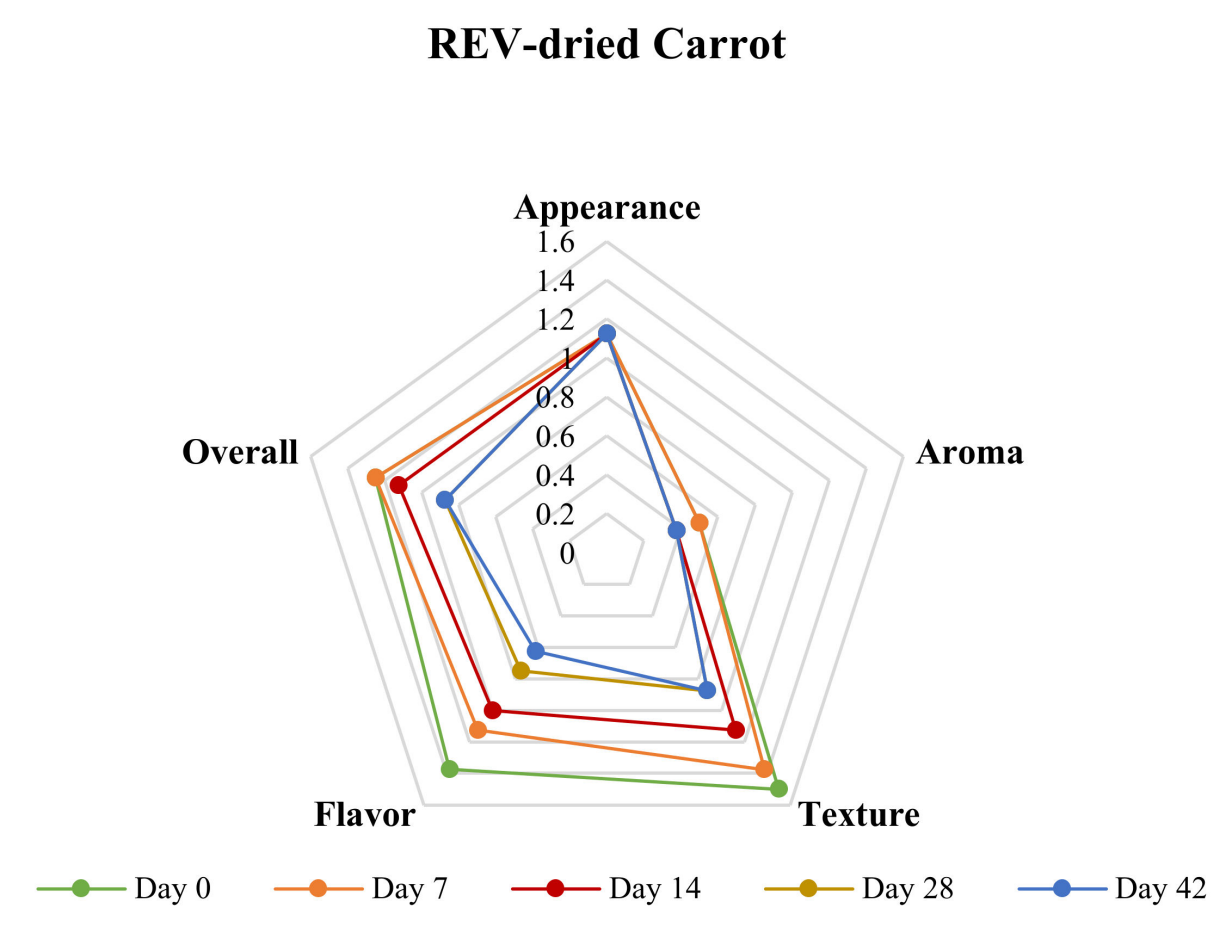
Cobweb diagram of five sensory attributes of REV-dried broccoli, orange, and carrot over the accelerated shelf life study based on panelists’ rating.

The sensory evaluation tests over the accelerated shelf-life study were performed on days 0, 7, 14, 28, and 42 after starting the experiment. The details of the scores of all five sensory attributes versus the storage date are exhibited in Figure 8. For REV-dried broccoli, as can be seen from the graph, the overall sensory attributes’ scores did not change in the accelerated shelf life. However, there were some slight fluctuations in the scores of aroma, texture, and flavor. This may be attributed to the panelists’ moods or health conditions at the date of the test. It could also be noticed that during the accelerated shelf-life, the scores of the appearance fell continuously. The relatively high temperature during storage may be the reason for the appearance change, since the degradation of chlorophyll in broccoli happens when it is exposed to a high storage temperature [37].

For REV-dried oranges, the scores almost did not change during the 42-day shelf life, and all stayed at a high level. This means that the sensory properties are well retained in oranges even if at elevated storage temperature and the oranges’ acidic nature may account for that [35].

As could be seen from the Figure, with the increase of storage time, the overall score for REV-dried carrots experienced a slow downward trend. This could be associated with color loss because of oxidation of highly unsaturated molecules, and the degradation of β-carotene, which can lead to the development of an off-flavor in dehydrated carrots [38]. However, all the attributes still obtained positive feedback from the panelists, which proved the acceptability of the products.

## 4. Conclusions

REV-drying is a rapid and advanced dehydration method for vegetables and fruits like broccoli, oranges, and carrots. It can significantly reduce the time of processing and better retain the nutritional values such as vitamin C and β-carotene compared to the conventional air-drying process. The rehydration potential showed to be the best with the freeze-dried samples, but the loss of volatiles during processing led the freeze-dried samples to lose scores in the sensory evaluation tests. Instead, the REV-dried products earned the highest scores in all the sensory properties, except texture for broccoli, used in this study (appearance, aroma, texture, flavor, overall). In the accelerated shelf-life study conducted on the REV-dried samples, the high temperature of storage may accelerate the deterioration rate of food samples, which in turn leads to a loss in the nutritional values.

## Figures and Tables

**Table 1:** Moisture content, water activity, and nutritional value of dried samples with different drying methods.

Treatment	Moisture Content (%) (*w/w*)	Water Activity	Carotene (µg/g Dry Weight)	Ascorbic Acid (mg/g)
Fresh Broccoli	88.8	-	-	3.79 ± 1.05 ^a^
Air-dried Broccoli	5.20 ± 0.15 ^a^	0.27 ± 0.01 ^a^	-	1.27 ± 0.27 ^b^
REV-dried Broccoli	5.80 ± 20 ^a^	0.31 ± 0.05 ^a^	-	3.61 ± 0.13 ^a^
Freeze-dried Broccoli	3.70 ± 0.56 ^b^	0.12 ± 0.01 ^b^	-	3.77 ± 0.06 ^a^
Fresh Orange	81.80	-		3.97 ± 0.23 ^a^
Air-dried Orange	9.13 ± 0.05 ^a^	0.31 ± 0.01 ^b^	-	2.65 ± 0.27 ^d^
REV-dried Orange	9.13 ± 0.32 ^a^	0.28 ± 0.01 ^c^	-	3.29 ± 0.09 ^c^
Freeze-dried Orange	6.66 ± 0.41 ^b^	0.36 ± 0.00 ^a^	-	3.82 ± 0.06 ^b^
Fresh Carrot	90.03	-	3.03	-
Air-dried Carrot	15.03 ± 1.60 ^a^	0.49 ± 0.04 ^a^	0.52 ± 0.05 ^c^	-
REV-dried Carrot	9.00 ± 0.36 ^b^	0.28 ± 0.01 ^b^	1.28 ± 0.26 ^b^	-
Freeze-dried Carrot	8.43 ± 0.41 ^b^	0.05 ± 0.01 ^c^	2.98 ± 0.09 ^a^	-

Various letters in the same column within the same section show a significant difference (*p* ≤ 0.05).

**Table 2:** Specific moisture extraction rate (SMER), specific energy consumption (SEC), and η_ex_ values for the REV-dried broccoli, oranges, and carrots.

Product	Microwave Power	SMER (kg/kWh)	SEC (MJ/kg)	η_ex_
Broccoli	600 W	4.415	0.815	55.75
	1 kW	1.962	1.835	24.78
	2 kW	1.536	2.344	19.39
Oranges	600 W	2.158	1.669	12.24
	1 kW	1.942	1.854	11.02
Carrots	600 W	4.203	0.857	39.04
	1 kW	3.303	1.090	30.69
	2 kW	2.202	1.635	20.46

**Table 3:** Reaction rate constant (k) and half-life ($t_{1/2}$) vitamin C loss in REV-dried broccoli and orange.

Product	Temperature (°C)	$t_{1/2}\;(\mathrm{Day})$	k (Day^−1^)	*R* ^2^
REV-dried broccoli	35	36.48	0.019	0.9772
REV-dried orange	35	46.21	0.015	0.9721
